# SWATH mass spectrometry as a tool for quantitative profiling of the matrisome

**DOI:** 10.1016/j.jprot.2018.02.026

**Published:** 2018-10-30

**Authors:** Lukas Krasny, Philip Bland, Naoko Kogata, Patty Wai, Beatrice A. Howard, Rachael C. Natrajan, Paul H. Huang

**Affiliations:** a Division of Molecular Pathology, The Institute of Cancer Research, London, SW3 6JB, UK; b The Breast Cancer Now Toby Robins Research Centre, Division of Breast Cancer Research, The Institute of Cancer Research, London, SW3 6JB, UK

**Keywords:** Matrisome, Extracellular matrix, SWATH MS, DIA MS, Proteomics, Mass spectrometry

## Abstract

Proteomic analysis of extracellular matrix (ECM) and ECM-associated proteins, collectively known as the matrisome, is a challenging task due to the inherent complexity and insolubility of these proteins. Here we present sequential window acquisition of all theoretical fragment ion spectra mass spectrometry (SWATH MS) as a tool for the quantitative analysis of matrisomal proteins in both non-enriched and ECM enriched tissue without the need for prior fractionation. Utilising a spectral library containing 201 matrisomal proteins, we compared the performance and reproducibility of SWATH MS over conventional data-dependent analysis mass spectrometry (DDA MS) in unfractionated murine lung and liver. SWATH MS conferred a 15–20% increase in reproducible peptide identification across replicate experiments in both tissue types and identified 54% more matrisomal proteins in the liver versus DDA MS. We further use SWATH MS to evaluate the quantitative changes in matrisome content that accompanies ECM enrichment. Our data shows that ECM enrichment led to a systematic increase in core matrisomal proteins but resulted in significant losses in matrisome-associated proteins including the cathepsins and proteins of the S100 family. Our proof-of-principle study demonstrates the utility of SWATH MS as a versatile tool for in-depth characterisation of the matrisome in unfractionated and non-enriched tissues.

**Significance:**

The matrisome is a complex network of extracellular matrix (ECM) and ECM-associated proteins that provides scaffolding function to tissues and plays important roles in the regulation of fundamental cellular processes. However, due to its inherent complexity and insolubility, proteomic studies of the matrisome typically require the application of enrichment workflows prior to MS analysis. Such enrichment strategies often lead to losses in soluble matrisome-associated components. In this study, we present sequential window acquisition of all theoretical fragment ion spectra mass spectrometry (SWATH MS) as a tool for the quantitative analysis of matrisomal proteins. We show that SWATH MS provides a more reproducible coverage of the matrisome compared to data-dependent analysis (DDA) MS. We also demonstrate that SWATH MS is capable of accurate quantification of matrisomal proteins without prior ECM enrichment and fractionation, which may simplify sample handling workflows and avoid losses in matrisome-associated proteins commonly linked to ECM enrichment.

## Highlights

• SWATH MS is capable of quantitative analysis of the matrisome in both non-enriched and ECM enriched tissues.
• SWATH MS provides a more reproducible coverage of the matrisome compared to DDA MS.
• Quantitative analysis by SWATH MS reveals that the lung has a higher matrisomal protein content versus the liver.
• Comparative analysis finds that ECM enrichment leads to significant losses in matrisome-associated proteins.

## Graphical abstract

**Figure:**
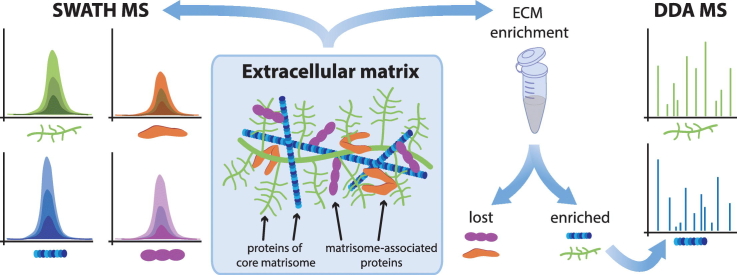

## 1 Introduction

The extracellular matrix (ECM) is a complex network of proteins whose primary scaffolding function confers integrity and elasticity to tissues and organs [1]. The ECM also provides important biochemical and biophysical cues required for the regulation of fundamental cellular processes including proliferation, differentiation, senescence and death [2]. The critical role of the ECM in human physiology is illustrated by a range of genetic diseases driven by dysregulation of key ECM components, such as osteogenesis imperfecta and Marfan syndrome [[3], [4], [5], [6], [7], [8]]. Accurate proteomic characterisation the ECM has historically been challenging due to a lack of consensus of the protein constituents that make up the ECM. To address this challenge, an effort to integrate proteomic and genomic datasets has led to the generation of a database of ECM and ECM-associated proteins known as the *in-silico* matrisome [9,10]. This matrisome database is divided into the “core matrisome” which consists of glycoproteins, collagens and proteoglycans subclasses, and the “matrisome-associated proteins” that comprise affiliated proteins, regulators and secreted factors [10]. It should be noted that the matrisome database was designed to be inclusive and contains matrisome-associated proteins that are predicted to interact with ECM components. In particular, the interaction of multiple proteins within the “secreted factors” subclass with the ECM remains to be experimentally confirmed.

Many components of the matrisome, in particular the core matrisomal proteins, are highly insoluble as a result of large size, extensive glycosylation and complex covalent crosslinking [11]. In addition, a number of matrisome-associated proteins are found in low abundance and may be obscured by the more abundant intracellular proteins. There has therefore been a need to develop ECM enrichment strategies that are capable of both solubilising matrisomal components and distinguishing these proteins from contaminating intracellular proteins that may otherwise dominate the acquired MS data in proteomic experiments [10,[12], [13], [14], [15]]. We and others have shown that while the majority of these strategies lead to an enrichment in core matrisome components, this is routinely accompanied by a concomitant decrease in the soluble matrisome-associated proteins [10,12,16]. The losses in matrisome-associated proteins stem from the multiple steps of enrichment, fractionation and washes associated with these ECM enrichment workflows. Such enrichment methods create a distorted view of the actual matrisomal content of the system under study, highlighting an urgent need for accurate methods capable of quantifying the matrisome with minimal enrichment and decellularisation of tissue specimens.

The development of sequential window acquisition of all theoretical fragment ion spectra (SWATH) or data-independent acquisition (DIA) mass spectrometry (MS) has led to a new era of accurate and reproducible label-free quantification of proteomes [17,18]. Unlike conventional data-dependent acquisition (DDA) MS which relies on the specific selection and fragmentation of a fixed number of (typically abundant) precursor ions in a survey scan, SWATH MS is based on the cyclical acquisition of precursor ions with fixed isolation windows that cover the entire *m*/*z* range. In this manner, all ionized precursor peptides within a sample are fragmented and their fragmentation spectra collected, enabling retrospective interrogation of the peptides of interest using spectral libraries [17]. SWATH MS combines the advantages of high reproducibility and sensitivity of targeted methods like selected reaction monitoring/multiple reaction monitoring (SRM/MRM) with the increased proteome depth typically seen with DDA MS [18,19]. SWATH MS is versatile and has been used in diverse applications including the quantification of proteins in a number of model organisms, diseases states and bacteria [[20], [21], [22], [23], [24], [25], [26], [27], [28]]. SWATH MS has also been useful in characterising of low abundance sub-proteomes including post-translational modifications such as acetylation, succinylation and glycosylation [[29], [30], [31], [32]].

Here we describe the first study to utilise SWATH MS as a tool for robust identification and quantification of matrisomal proteins. By employing a spectral library containing 201 mouse matrisomal proteins, we show that this strategy outperforms DDA by providing a more comprehensive and reproducible coverage of the matrisome while dispensing with the need for extensive fractionation in murine lung and liver tissue. We demonstrate that SWATH MS is capable of measuring both non-enriched and ECM enriched tissue lysates and use this method to highlight the scale of losses in matrisome-associated proteins resulting from ECM enrichment. This study illustrates the utility of SWATH MS as a valuable alternative for deep characterisation of matrisomal proteins from complex non-enriched tissues.

## 2 Material and methods

### 2.1 Animal models and tissue collection

All animal work was carried out under UK Home Office project and personal licenses following local ethical approval from The Institute of Cancer Research Ethics Committee and in accordance with local and national guidelines. 6 liver and 6 lung tissue samples were dissected from 14-week-old to 18-week-old virgin female SCID Beige mice. Tissue samples were snap frozen in liquid nitrogen directly after excision and stored at −80 °C.

### 2.2 Tissue processing

Tissue samples were cut into small pieces, weighed and placed into precooled tubes containing PBS solution with 10 KIU/ml aprotinin (Sigma). Samples were washed for 30 min at 4 °C to remove excess blood. Samples were then transferred into homogenization buffer at 4 ml per g of tissue. The homogenization buffer is comprised of Tris-HCl (50 mM, pH 7.4), 0.25% 3-[(3-Cholamidopropyl)dimethylammonio]-1-propanesulfonate hydrate (CHAPS, Sigma), 25 mM EDTA, 3 M NaCl (Sigma) and 10 KIU/ml aprotinin. Tissue was homogenized 2 × 30 s on ice with a LabGEN700 homogenizer. The homogenized tissue was placed on a rotator (20 rpm) for 20 min at 4 °C. The resulting homogenate was split and subjected to one of two procotols as follows:

a) Non-enriched samples: 1/3 of the homogenate was subjected to acetone precipitation by mixing with 4 volumes of ice cold acetone, vortexed and incubated 2 h at −20 °C to precipitate proteins. The precipitate was spun for 15 min at 15,000 rpm, 4 °C and the supernatant removed. The resulting pellet was resuspended in 0.3 ml of urea buffer consisting of 8 M urea, 100 mM ammonium bicarbonate (ABC, Sigma), 25 mM tris(2-carboxyethyl)phosphine (TCEP, Sigma) and stored at −80 °C.
b) ECM enrichment: The remaining 2/3 of the homogenate was subjected to ECM enrichment as previously described by Hill et al. [12] with minor changes. The homogenate was spun for 15 min at 15,000 rpm, 4 °C, and the supernatant was removed. The pellet was resuspended in 0.5 ml of the fresh homogenization buffer and washed for two additional times in this buffer on a rotator (20 rpm) for 20 min at 4 °C. The CNBr digestion step of the original protocol was not performed and instead, the pellet after the third wash was directly resuspended in 0.3 ml of urea buffer and stored at −80 °C.

The samples used for generation of the spectral library were not split after homogenization and the acetone precipitation protocol was applied to the whole tissue. Final protein concentration of all samples after resuspension in urea buffer was measured using the Pierce 660 nm Protein assay as per manufacturer's instructions (Thermo Scientific).

### 2.3 Protein digestion and sample preparation

400 μg of protein for spectral library generation or 100 μg of protein for all other experiments was reduced with 20 mM dithiothreitol (DTT, Sigma), at 56 °C for 40 min and alkylated with 30 mM iodoacetamide (IAA, Sigma) at 25 °C for 25 min in the dark. To remove CHAPS, gel-assisted digestion was performed as described previously [33]. Briefly, a mixture of 30% (v/v) acrylamide/bisacrylamide (29:1), 10% (w/v) APS and TEMED was added directly to the tube with alkylated proteins and allowed to polymerize in air at room temperature. The resulting gel was cut into small cubes and sequentially dehydrated with 50% ACN in 50 mM ABC and 100% ACN. Compared to original protocol [33], a solution with twice the concentration of trypsin (25 ng/ml in 100 mM ABC) was added to the dry gel cubes and incubated for 30 min at 4 °C followed by addition of 100 mM ABC to suspend all the gel cubes. Proteins were left to digest overnight at 37 °C. After digestion, samples were spun for 15 min at 15,000 rpm, 4 °C, and the supernatant with digested peptides was removed. The gel cubes were subjected to three additional extractions for 10 min in 60% ACN, 0.1% TFA. Pooled supernatant and extracts were dried in a SpeedVac concentrator and desalted with OPTI trap cartridges (Optimize Technologies).

### 2.4 Strong cation exchange (SCX) chromatography

Tryptic digests from non-enriched sample preparations of 2 lung and 2 liver samples were selected for the generation of the spectral library. 400 μg of the digested samples were resuspended in 20 μl of Buffer A (10 mM KH_2_PO_4_ in 20% ACN, pH 2.65) and loaded on a 2.1 × 100 mm Polysulfoethyl A column with 5 μm, 200 Å particles (PolyLC Inc.) for SCX fractionation. The peptides were eluted at a flow rate of 0.2 ml/min by Buffer B (10 mM KH_2_PO_4_, 500 mM NaCl in 20% ACN, pH 2.65) with a gradient of 0–10% of B for 2.5 min, 10–50% of B for 20 min, 50–100% B for 7.5 min followed by an additional 10 min elution with 100% B. 12 fractions were collected over 39 min with fraction 1 collected from 0 to 12 min and fraction 12 from 32 to 39 min. The remaining 10 fractions were collected at 2 min intervals between 12 and 32 min. All SCX fractions were dried in a SpeedVac concentrator, then resuspended in 0.1% TFA and desalted with C18 OMIX tips (Agilent Technologies).

### 2.5 DDA MS data acquisition

For liquid chromatography-tandem mass spectrometry (LC-MS/MS) analysis, non-enriched unfractionated samples and SCX fractions were dissolved in Buffer A (2% ACN, 0.1% FA), spiked with iRT calibration mix (Biognosys AG) and analysed on an Agilent 1260 HPLC system coupled to a TripleTOF 5600+ mass spectrometer with NanoSource III (AB SCIEX). 1 μg of peptides for each sample was loaded onto a ZORBAX C18 (Agilent Technologies) trap column and separated on a 75 μm × 15 cm long analytical column with an integrated manually pulled tip packed with Reprosil Pur C18AQ beads (3 μm, 120 Å particles, Dr. Maisch) with a linear gradient of 2–40% of Buffer B (98% ACN, 0.1% FA) in 90 min and a flow rate of 250 nl/min. Full profile MS scans were acquired in the mass range of *m*/*z* 340–1500 in positive ion mode. The top 20 most intense ions with charge state from 2^+^ to 5^+^ were selected for fragmentation and MS/MS scans were acquired in mass range of *m*/*z* 100–1500. Maximum filling time for MS scans was 250 ms, for MS/MS scans 100 ms and dynamic exclusion of fragmented ions was set to 12 s.

### 2.6 SWATH MS data acquisition

SWATH MS data for both non-enriched and enriched unfractionated samples were acquired on the same MS instrument using the identical LC conditions. Full profile MS scans were acquired in mass range of *m*/*z* 340–1400 in positive ion mode. As previously described by Bruderer et al. [34], 8 data points per elution peak were set-up for calculation of 31 precursor isolation windows with a fixed size of 25 Da across the mass range of *m*/*z* 350–1250 with 1 Da overlap. MS/MS scans were acquired in the mass range of *m*/*z* 100–1500. Maximum filling time for MS scans was 250 ms and for MS/MS scans 100 ms, resulting in a cycle time of 3.3 s.

### 2.7 Spectral library generation

The 2 liver and 2 lung samples that were fractionated by SCX were used for building the spectral library. Acquired DDA datasets were searched by ProteinPilot 5.0.1 software (AB SCIEX) against a Uniprot mouse database (downloaded on 20/10/2017) with added iRT sequence. Each dataset comprised of 12 DDA runs for 12 individual SCX fractions, which were combined during the search. Overall 4 datasets were searched, 2 each for liver and lung. The Spectronaut 11 software (Biognosys AG) [19] was used to generate four spectral libraries from the resulting ProteinPilot group files using following settings: maximum 2 missed cleavages, carbamidomethylation of cysteines set as fixed modification, asparagine and glutamine deamidation, oxidation of methionine and hydroxylation of lysine and proline were set as variable modifications, precursor charge state from 2^+^ to 5^+^, 99% confidence of the correct peptide identification. Other parameters were used in default settings. The generated libraries were then merged into a final spectral library containing 41,741 precursors from 5050 protein groups.

### 2.8 Data processing and statistical analysis

DDA data for non-enriched samples were searched by ProteinPilot 5.0.1 software (AB SCIEX) against a Uniprot mouse database (downloaded on 20/10/2017) with added iRT sequences. The search parameters were as follows: maximum 2 missed cleavages, carbamidomethylation of cysteines set as fixed modification, biological modification, urea denaturation and collagen emphasis in ProteinPilot were selected as variable modifications. For comparison with SWATH, only peptides with 99% confidence of the correct identification (with an average peptide-to-spectrum match false discovery rate (PSM-FDR) [35] of 1%) and proteins with a (protein-to-peptide) FDR below 5% were selected for analysis.

The acquired SWATH data were analysed using the Spectronaut 11 software with the default analysis settings and the following minor changes: FDR of protein identification was set to 5% (with PSM-FDR threshold maintained at 1%) and maximum number of 6 precursors was used for quantification. Peak area of fragment ions was used for peptide quantification. To quantify proteins, the mean value of the peptides quantities was calculated. Results of protein quantification were exported as a tsv file and further processed and statistically analysed in Perseus (ver. 1.5.6.0) [36]. All data were log2 transformed and the differences in protein levels were compared by Welch's *t*-test with Benjamini's-Hochberg's correction of *p*-value [35]. The resulting q-value of 0.1 was set as a cut-off. Datasets were further *Z*-normalized and hierarchically clustered based on Euclidean distance.

The MS data has been deposited to the ProteomeXchange Consortium via the PRIDE [37] partner repository with the dataset identifier PXD008651.

## 3 Results

### 3.1 Spectral library generation

**Fig. 1:**
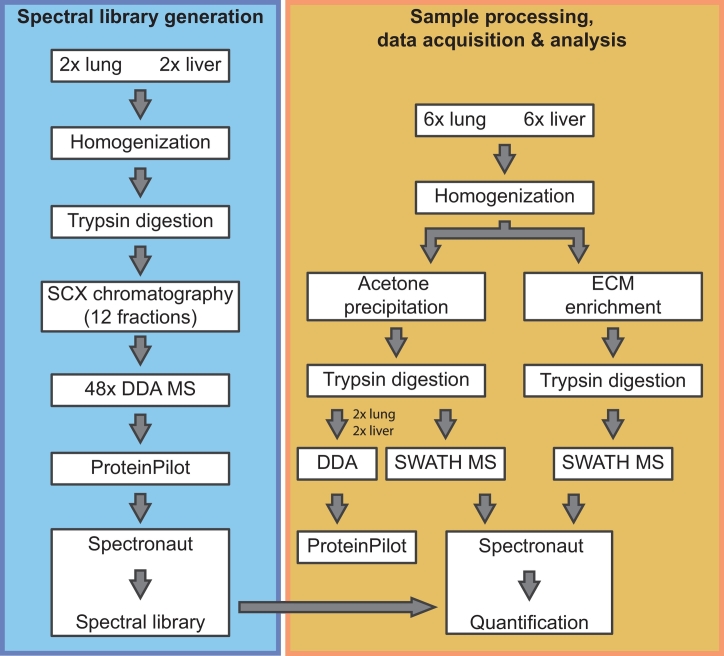
Schematic of the experimental workflow employed in this study. Key steps that were undertaken for the generation of the spectral library (blue) and sample processing, data acquisition and analysis (orange) in this study are highlighted. (For interpretation of the references to color in this figure legend, the reader is referred to the web version of this article.)

Spectral libraries are required for effective post-acquisition deconvolution and processing of SWATH MS data [17]. These spectral libraries contain parameters such as *m*/*z* value, intensities and retention times for all precursors and their respective ion fragments (transitions), which are extracted from prior discovery DDA MS experiments [38]. There is no publicly available spectral library for *Mus musculus* on SWATHAtlas (www.swathatlas.org) and we sought to first generate such a library based on mouse liver and lung tissue homogenates. Tissue homogenates were subjected to SCX fractionation in 2 biological replicates and each of the 12 fractions run in DDA mode with spiked indexed retention time calibration (iRT) peptides. The Spectronaut 11 software [19] was used to combine the data from the 4 experiments (2 replicates each of liver and lung) to generate a spectral library containing 41,741 precursors in 5,050 protein groups, including 201 matrisomal protein (Table 1, Supplemental Table S1). The scheme of the workflow for spectral library generation as well as for sample processing is depicted in Fig. 1.

### 3.2 Comparison of DDA and SWATH MS matrisome analysis

As a result of stochastic precursor selection and fragmentation, DDA MS suffers from a well-documented shortcoming of poor reproducibility in the identification (ID) of peptides between different experiments [39]. In contrast, since the fragmentation ions for all peptide precursors in the scanned mass range are acquired in a SWATH MS experiment, this strategy has previously been shown to significantly enhance reproducibility in peptide IDs [18,19,40]. To ascertain if this increased reproducibility is also applicable to the matrisome, we performed a comparative analysis of the performance of DDA versus SWATH MS in mouse liver and lung tissues.

**Table 1:** Number of matrisomal proteins in the spectral library. More details can be found in Supplemental Table S1.

Matrisomal class	Number of proteins in spectral library
Glycoproteins	62
Collagens	14
Proteoglycans	9
Affiliated proteins	37
Regulators	59
Secreted factors	20
Total	201

**Fig. 2:**
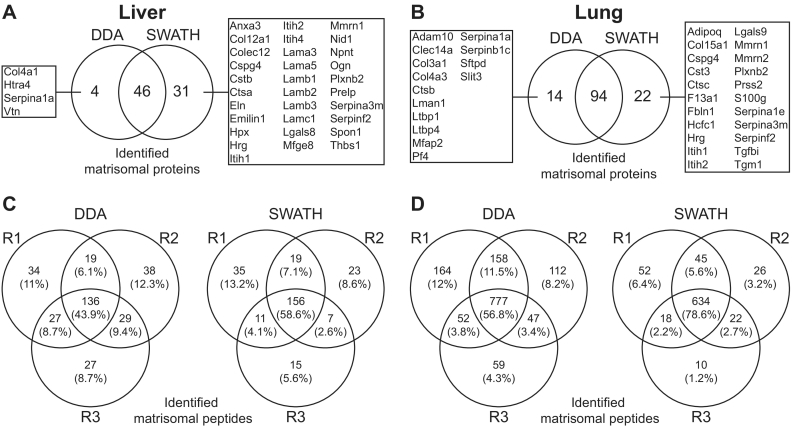
Comparative analysis of the performance of SWATH MS versus DDA MS. Venn diagrams depicting number and overlap of matrisomal proteins detected by DDA and SWATH MS in A) liver and B) lung tissue across 2 biological replicates and 3 technical replicates. Proteins uniquely identified by each method are highlighted. Venn diagrams depicting the number and overlap of unique matrisome peptides identified in C) liver and D) lung tissue across 3 technical replicates (R1-R3) in DDA and SWATH MS. Only peptides with 99% confidence of identification or higher were compared.

We first determined the number of matrisome proteins that were reproducibly identified in two biological replicates of unfractionated mouse liver and lung (each biological replicate was measured in three technical replicates). To avoid any bias associated with ECM enrichment pre-processing steps, we performed this analysis using non-enriched tissue lysates. DDA analysis of the liver samples resulted in the ID of 50 matrisomal proteins (Table 2, Supplemental Table S2), while SWATH MS analysis of identical samples identified 77 matrisomal proteins. 46 proteins were common between the two methods with 4 matrisomal proteins identified exclusively by DDA and 31 proteins identified exclusively by SWATH (Fig. 2A). In the lung tissue, 108 and 116 matrisomal proteins were identified by DDA and SWATH MS respectively (Table 2, Supplemental Table S2), with 14 matrisomal proteins identified only by DDA compared to 22 matrisomal proteins exclusively detected by SWATH MS (Fig. 2B). With a 54% increase in the number of protein IDs, our data suggests that in the context of the liver, SWATH MS is superior to DDA for matrisomal protein ID. However, the number of proteins identified in the lung by both DDA and SWATH MS is comparable. This data indicates that the benefit of SWATH over DDA MS in the number of unique matrisomal protein IDs may be tissue dependent.

To assess the reproducibility of peptide IDs between both methods, we compared the data from three technical replicates of the same liver and lung samples. DDA identified a higher total number of unique matrisomal peptides versus SWATH MS (Fig. 2C and D). However, in both the liver and lung tissue, SWATH MS outperforms DDA in the reproducibility of peptides identified across all three technical replicate analyses. Reproducibility of SWATH MS in the liver was 58.6% while DDA was only 43.9% (Fig. 2C). Similarly in lung, reproducibility of SWATH MS was 78.6% compared to 56.8% obtained by DDA (Fig. 2D). This 15–20% improvement agrees with previous reports that SWATH MS is superior to DDA in run-to-run reproducibility in peptide IDs [18,19,40].

**Table 2:** Number of identified matrisomal proteins in mouse liver and lung tissue by DDA versus SWATH MS. More details can be found in Supplemental Table S2.

	Liver		Lung	
	DDA	SWATH	DDA	SWATH
Glycoproteins	7	20	38	39
Collagens	9	9	11	10
Proteoglycans	4	6	7	7
Affiliated proteins	10	16	20	20
Regulators	20	26	25	32
Secreted factors	0	0	7	8
Total	50	77	108	116

### 3.3 Quantitative comparison of the lung and liver matrisome by SWATH MS

A further benefit afforded by SWATH over DDA MS is the ability to perform more precise label free quantification by comparing of signal intensities or peak areas of peptide-specific transitions [17]. We have previously undertaken a DDA MS-based comparison of murine liver and lung and demonstrated that while both organs share a common subset of matrisome components, the lung contains more unique matrisomal proteins versus the liver [16]. However, our previous study was qualitative in nature, limiting our ability to make conclusions about the quantitative differences in matrisomal content that is common to both organs. In addition, we cannot rule out the possibility that the matrisomal proteins found exclusively in the lung was not due to missed peptide precursors resulting from stochastic precursor ion selection in the DDA MS analysis of the liver.

**Fig. 3:**
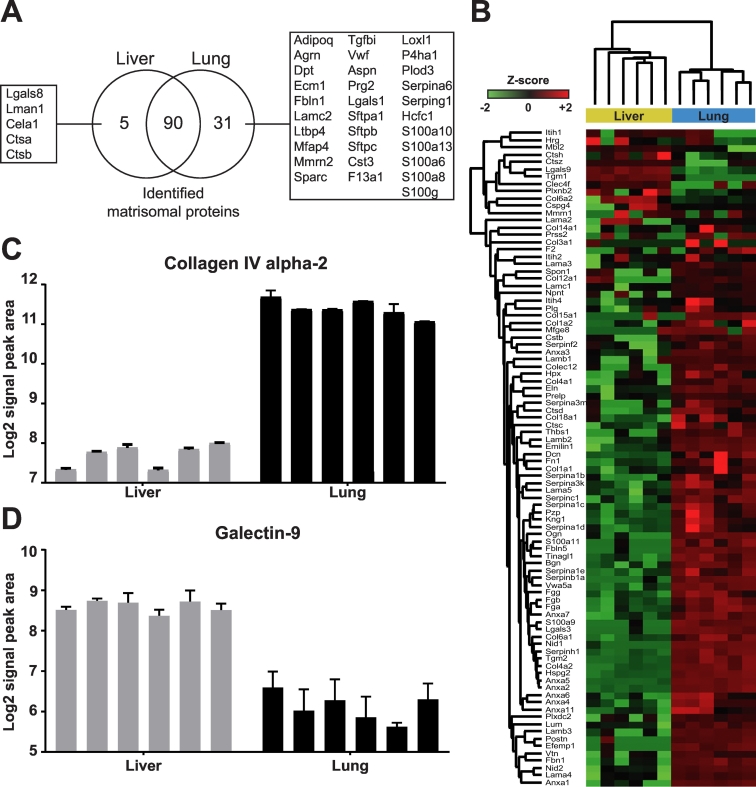
Comparative analysis of the liver and lung matrisome by SWATH MS. A) Venn diagram depicting the number and overlap of unique matrisomal proteins quantified by SWATH MS in liver and lung tissue across 6 biological replicates. B) A heatmap of the 90 common matrisomal proteins that were quantified in both liver and lung tissue (*n* = 6). Samples were subjected to two-way hierarchical clustering based on Euclidian distance. Relative quantification of C) Collagen IV and D) Galectin-9 in liver and lung tissue across the 6 biological replicates. Mean Log2 signal peak area from 3 technical replicates of each sample is shown with the standard deviation.

**Table 3:**
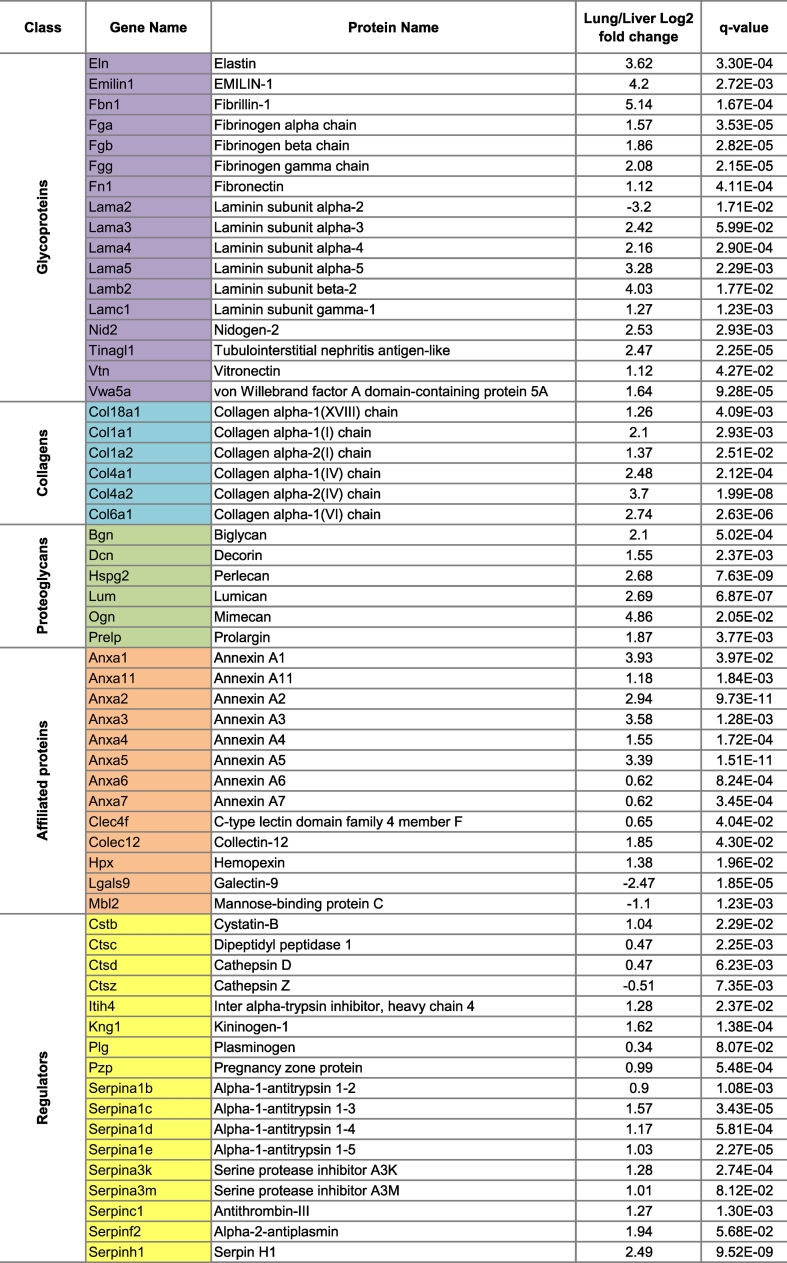
List of matrisomal proteins identified in both lung and liver tissue, whose expression levels are significantly altered.

Analysis of 6 non-enriched liver and lung samples (each measured in three technical replicates) resulted in the ID of 126 matrisomal proteins, 95 proteins of which were found in the liver and 121 in the lung (Fig. 3A, Supplemental Table S3). Of these proteins, 31 and 5 proteins were unique to the lung or liver respectively. As expected, the lung surfactant lipoproteins Sftpa1, Sftpb and Sftpc (affiliated proteins) were among the proteins found only in the lung. Other proteins exclusively identified in the lung include the glycoproteins Agrin (Agrn) and von Willebrand factor (Vwf), and the proteoglycan Asporin (Aspn). Absence of these proteins in liver matrisome is consistent with our previously published data [16]. Of the 90 matrisomal proteins that were common in both tissue types (Fig. 3B), 59 proteins were found to be significantly altered between the lung and liver (Table 3). The majority of these matrisomal proteins (55 proteins) were significantly increased in lung and include the important structural proteins Elastin (Eln, log2 fold change 3.62,) and Collagen IV (Col4a2, log2 fold change 3.7) (Fig. 3C). Only 4 proteins were found to be significantly increased in liver compared to lung. These are Laminin subunit alpha-2 (Lama2, log2 fold change 3.2), Mannose binding protein C (Mbl2, log2 fold change 1.1), Cathepsin Z (Ctsz, log2 fold change 0.51) and Galectin 9 (Lgals9, log2 fold change 2.47) (Fig. 3D). Our analysis confirms previous findings that the lung contains significantly higher levels of matrisomal proteins versus the liver [16] and highlights the ability of SWATH MS to perform quantitative comparisons of the matrisomal content between different tissue types in unfractionated and non-enriched tissue.

### 3.4 SWATH MS as a tool for characterising the matrisomal alterations associated with ECM enrichment

In our previous study, we performed a qualitative comparison of four ECM enrichment protocols on matrisome protein ID in four different murine tissue types and showed that all four ECM enrichment methods led to significant losses in soluble matrisome-associated proteins [16]. Here, we applied SWATH MS to quantitatively characterise the effects of ECM enrichment on matrisomal protein content by comparing non-enriched versus ECM-enriched liver and lung tissue. ECM was enriched by utilising a method originally developed by Hill et al. [12] with minor alterations. This method involves the use of a chemical digestion step by CNBr to aid in the solubilisation of the urea-insoluble ECM fraction prior to trypsin digestion. Given that the samples used for the generation of the spectral library were not digested by CNBr, we excluded this step from the working protocol. Instead, samples were digested after resuspension in urea buffer using an increased concentration of trypsin.

**Fig. 4:**
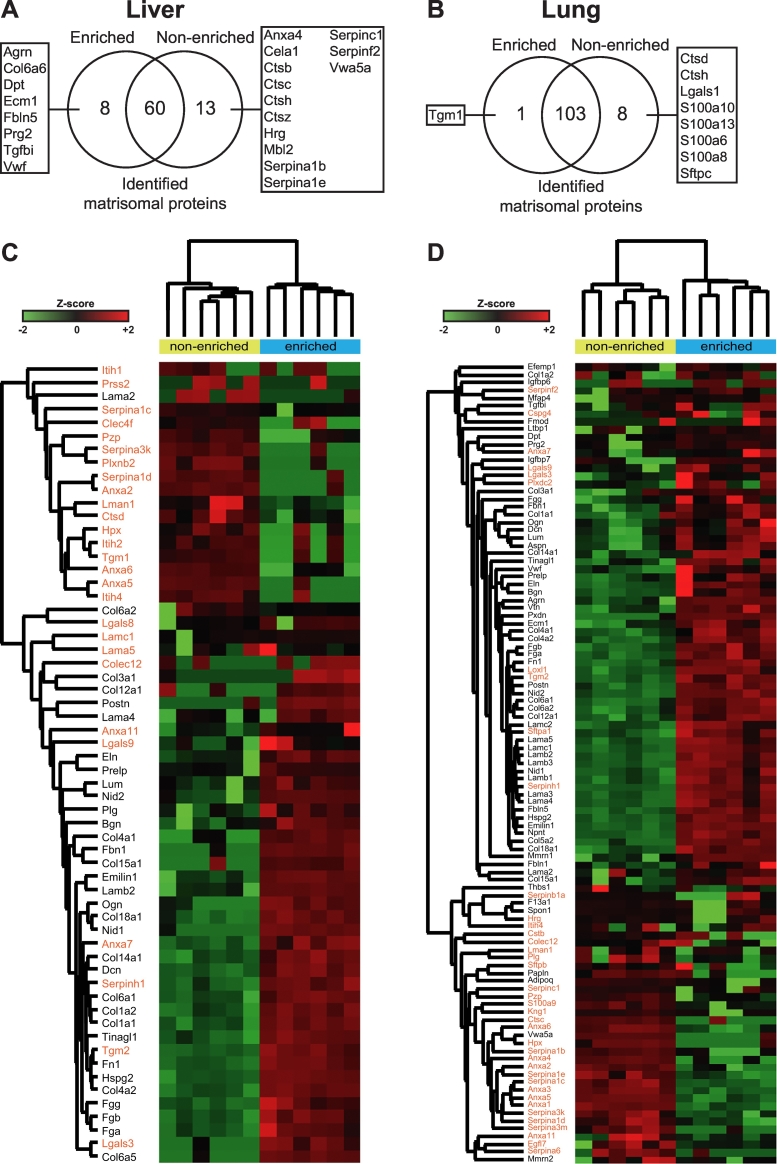
Effect of ECM enrichment on the matrisomal content in liver and lung as measured by SWATH MS. Venn diagram depicting the number and overlap of unique matrisomal proteins in ECM enriched versus non-enriched samples in A) liver and B) lung tissue across 6 biological replicates. Heatmaps of the common matrisomal proteins that were quantified in both ECM enriched and non-enriched samples in C) liver and D) lung tissue (*n* = 6). Samples were subjected to two way hierarchical clustering based on Euclidian distance. Core matrisomal proteins (black text) and matrisome associated proteins (orange text) are highlighted to illustrate gains in core matrisome proteins and losses in matrisome-associated proteins as a result of ECM enrichment. (For interpretation of the references to color in this figure legend, the reader is referred to the web version of this article.)

In the liver tissue, we quantified 68 matrisomal proteins in ECM enriched samples and 73 proteins in the non-enriched samples (Fig. 4A, Supplemental Table S4). 8 matrisomal proteins were found only in enriched samples and include 6 glycoproteins, 1 collagen and 1 proteoglycan; all proteins belonging to the core matrisome class. In contrast, 13 proteins primarily composed of the matrisome-associated class (2 affiliated proteins, 10 regulators and 1 glycoprotein) were detected exclusively in the non-enriched samples. 4 cathepsins (regulators) and 4 serpins (regulators) were lost during the enrichment. Among the 60 matrisomal proteins quantified in both sample groups (Fig. 4B), 35 were found to be significantly altered (Table 4). As expected, the vast majority of matrisome proteins showed a significant upregulation in the ECM enriched compared to the non-enriched samples (Table 4). Of the 31 matrisomal proteins that were upregulated in the enriched samples, 24 belong to the core matrisome with EMILIN-1 (Emilin1) and Mimecan (Ogn) showing the highest increase with a log2 fold change of 5.08 and 5.01, respectively. 4 matrisome-associated proteins were significantly decreased after enrichment, specifically Annexin A6 (Anxa6), Cathepsin D (Ctsd), Protein ERGIC-53 (Lman1) and Alpha-1-antitrypsin 1–3 (Serpina1c). The largest decrease was found in Anxa6, whose levels in enriched samples was almost 5 times lower (log2 fold change −2.24) compared to the non-enriched samples.

In the SWATH analysis of the lung specimens, 104 and 111 matrisomal proteins were identified in the enriched and non-enriched samples, respectively (Fig. 4C, Supplemental Table S5). Protein transglutaminase K (Tgm1, affiliated protein) was the only unique protein in the enriched samples while 8 matrisome-associated proteins (2 affiliated proteins, 2 regulators, 4 secreted factors) were found exclusively in the non-enriched lungs (Fig. 4C). Similar to the liver, ECM enrichment resulted in the loss of 2 cathepsins as well as 4 members of the S100 protein family (secreted proteins). Of the 103 proteins common between the two sample preparation workflows (Fig. 4D), 71 matrisomal proteins were significantly altered (Table 5). Similar to the liver samples, ECM enrichment resulted in the increase of a large proportion of the altered matrisomal proteins. Of the 53 matrisomal proteins that are significantly increased, 45 belong to the core matrisome. However, ECM enrichment also led to the decrease of 16 matrisome-associated proteins, including 7 of 8 identified Annexins (affiliated proteins), all identified Alpha-1-antitrypsins (regulator) and Epidermal growth factor-like protein 7 (Egfl7, secreted factor).

Our comparative analysis demonstrates that ECM enrichment significantly alters the matrisome composition identified in proteomic experiments with a general increase in core matrisomal components and a reduction in the levels of matrisome-associated proteins.

**Table 4:**
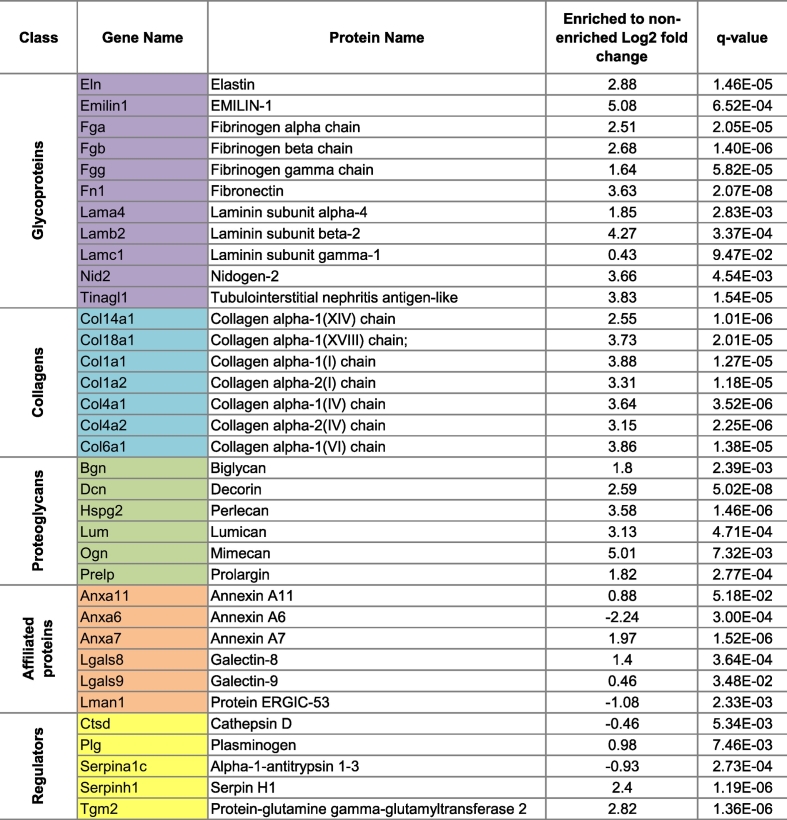
List of liver matrisomal proteins identified in both ECM enriched and non-enriched samples, whose levels were significantly altered by enrichment.

**Table 5:**
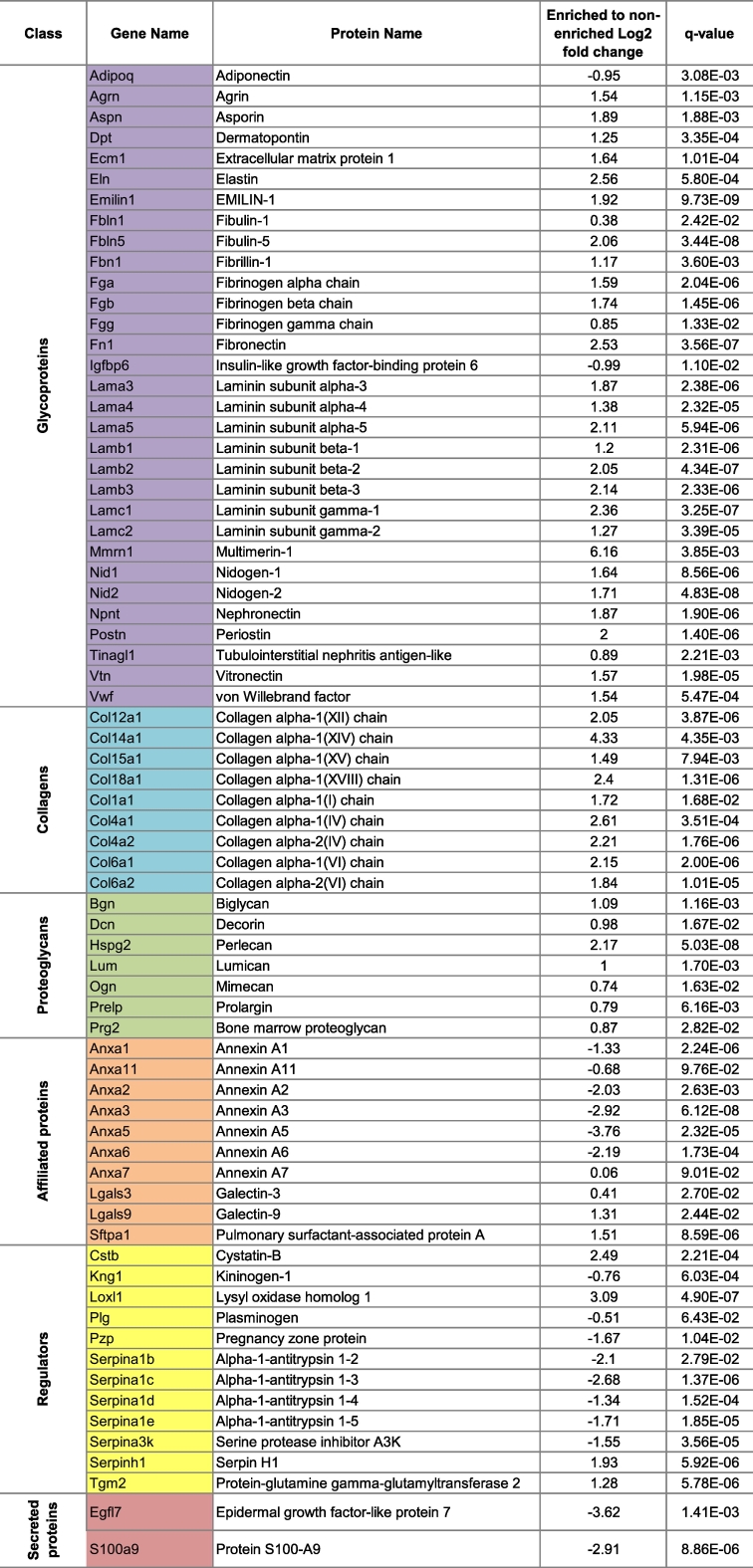
List of lung matrisomal proteins identified in both ECM enriched and non-enriched samples, whose levels were significantly altered by enrichment.

## 4 Discussion

In this study, we have provided the first proof-of-principle application of SWATH MS in the quantitative characterisation of matrisomal proteins. This involves the development of a murine specific spectral library comprising 201 matrisomal proteins across all six subclasses in both core matrisome and matrisome-associated proteins. We demonstrate the general applicability of this approach in measuring the matrisomal content in two different tissue types as well as in both non-enriched and ECM-enriched lysates. We anticipate that with further refinements in spectral library generation and the use of other species-specific spectral libraries, this strategy can be readily extended to studying different disease states in multiple organisms.

While DDA MS has been the foundation of modern proteomics for more than two decades, we show that SWATH MS confers three major advantages in the characterisation of the matrisome. The first key advantage is increased reproducibility between experiments, as SWATH MS does not suffer from the drawback of precursor ion undersampling in complex samples routinely found in DDA MS experiments. In line with previously published SWATH studies [18,19,40], our data demonstrates that SWATH MS increases the reproducibility of peptide IDs by 15–20% across multiple technical replicates. The second advantage of SWATH MS is a more comprehensive characterisation of the liver matrisome where a 54% increase in protein IDs was observed compared to DDA MS of the same samples. Unlike DDA MS, SWATH is not dependent on precursor ion selection and fragments all ions in pre-defined isolation windows. Therefore, even precursors with low signal intensities are fragmented and fragmentation ion spectra acquired. This increased benefit was not observed in the context of the lung which suggests that SWATH analysis may be particularly useful in tissues with low ECM content such as the liver where low intensity matrisomal peptide precursors may be obscured by the more abundant intracellular proteins and missed in DDA mode [41]. Alternatively, distinct patterns of post-translational modifications (PTMs) in matrisomal proteins (such as glycosylation) between the liver and lung may skew the DDA MS protein ID rates. Future work incorporating PTMs into the spectral library will enable a more comprehensive interrogation of the basis for the observed tissue dependent benefit of SWATH MS. Finally, SWATH MS enables precise label-free quantification and we exploit this property to accurately quantify matrisomal alterations between two tissue types and compare distinct sample preparation workflows.

There are some limitations to the SWATH MS strategy. As with all targeted MS approaches, the number of proteins identified in a SWATH MS analysis is largely limited by the composition of the spectral library. Our spectral library consists of 201 matrisomal proteins which is only a fraction of the reported proteins found in the *in silico* matrisome database [10]. Future work will involve significantly increasing the spectral library by incorporating further discovery-based DDA analysis of additional murine tissue and cell types. It should be noted that since existing SWATH MS datasets can be retrospectively analysed with new spectral libraries, there is no requirement for additional experiments to re-acquire MS data. Furthermore, our spectral library is currently limited to trypsin digested peptides which restricts our analysis to urea soluble ECM components. With advances in sample preparation techniques including the use of hydroxylamine as a means to chemically digest insoluble ECM components [42], we anticipate that the depth of matrisome coverage can be readily improved by generating spectral libraries that include chemical digested peptides. Another limitation is that it is currently not possible to perform multiplex experiments in SWATH MS, restricting its application in large-scale experiments that have been previously reported with matrisomal analysis using DDA-specific isobaric chemical labelling strategies such as iTRAQ or TMT [[43], [44], [45]].

The majority of published matrisomal proteomics studies have employed extensive fractionation and/or ECM enrichment in order to enhance the number of proteins identified. We demonstrate the utility of SWATH MS in identifying a comparable number of matrisomal proteins in non-enriched unfractionated samples. For instance, Gocheva et al. [43] very recently identified 113 matrisomal proteins in murine lung in a workflow that included ECM-enrichment and off-gel fractionation. This number is comparable with the 121 matrisomal proteins we identified in our SWATH MS analysis of non-enriched unfractionated lungs. By quantitatively measuring the changes in matrisomal proteins associated with ECM enrichment, we further show that these workflows result in a systematic bias where a gain in core matrisome proteins is accompanied by losses in matrisome-associated proteins. Such losses in matrisome-associated proteins arising from ECM enrichment have previously been documented [12,16]. Our analysis finds that in some cases, specific matrisome-associated proteins such as the cathepsins and members of the S100 protein family are completely lost, highlighting a subset of the matrisome which is inaccessible by current ECM enrichment workflows; and may only be amenable to analysis by alternative approaches such as SWATH MS or ultra-deep proteomic sequencing of non-enriched samples. However, as with all proteomic enrichment strategies, these losses need to be balanced with any requirements for improved detection and quantification of low abundance as well as insoluble core matrisome proteins afforded by the use of ECM enrichment.

## 5 Conclusion

In summary, our current study finds that the SWATH MS strategy is a versatile tool for the quantitative analysis of non-enriched and ECM enriched tissue lysates without the need for prior fractionation. We show that this approach is capable of accurately quantifying matrisomal proteins in non-enriched tissue to a similar depth as ECM-enriched lysates, indicating that this methodology may reduce the requirements for extensive ECM enrichment in future studies, not only simplifying sample handling, but also avoiding associated losses in matrisome-associated proteins.

The following are the supplementary data related to this article.

Supplemental Table S1 Complete list of the murine matrisomal proteins included in the generated SWATH spectral library. Proteins are classified by matrisome categories (column A). Supplemental Table S1

Supplemental Table S2 List of individual matrisomal proteins identified by DDA MS and SWATH MS across 2 non-enriched samples of liver and lung. Proteins are classified by matrisome categories (column A). Supplemental Table S2

Supplemental Table S3 Quantitative comparison of 125 matrisomal proteins identified across lung and liver samples (*n* = 6). Proteins are classified by matrisome categories (column A) and ordered alphabetically within each category. Mean Log2 values of signal peak area for both liver and lung samples are shown in columns E and F, respectively. Log2 fold change for each protein is given in column G. Difference in mean Log2 values between liver and lung samples was tested by Welch's *t*-test with Benjamini's-Hochberg's correction of *p*-value. Statistically significant difference (q < 0.01) in Log2 values and calculated q-values are shown in column H and I, respectively. “NaN” indicates missing or incalculable values. Supplemental Table S3

Supplemental Table S4 Quantitative comparison of 81 matrisomal proteins identified across ECM enriched and non-enriched liver samples (*n* = 6). Proteins are classified by matrisome categories (column A) and ordered alphabetically within each category. Mean Log2 values of signal peak area for both ECM enriched and non-enriched liver samples are shown in columns E and F, respectively. Log2 fold change for each protein is given in column G. Difference in mean Log2 values between ECM enriched and non-enriched samples was tested by Welch's *t*-test with Benjamini's-Hochberg's correction of *p*-value. Statistically significant difference (q < 0.01) in Log2 values and calculated q-values are shown in column H and I, respectively. “NaN” indicates missing or incalculable values. Supplemental Table S4

Supplemental Table S5 Quantitative comparison of 112 matrisomal proteins identified across ECM enriched and non-enriched lung samples (*n* = 6). Proteins are classified by matrisome categories (column A) and ordered alphabetically within each category. Mean Log2 values of signal peak area for both ECM enriched and non-enriched lung samples are shown in columns E and F, respectively. Log2 fold change for each protein is given in column G. Difference in mean Log2 values between ECM enriched and non-enriched samples was tested by Welch's *t*-test with Benjamini's-Hochberg's correction of *p*-value. Statistically significant difference (q < 0.01) in Log2 values and calculated q-values are shown in column H and I, respectively. “NaN” indicates missing or incalculable values. Supplemental Table S5

## Author contribution

L.K., P.B., N.K., P.W., B.A.H. and R.C.N. performed the research; L.K. and P.H.H. analysed the data and wrote the paper with input from all the authors.

## Conflict of interest

The authors declare no competing financial interests.

## Transparency Document

Transparency document. Image 1
